# A Systematic Review of Topic Modeling Techniques for Electronic Health Records

**DOI:** 10.3390/healthcare14020282

**Published:** 2026-01-22

**Authors:** Iqra Mehmood, Zoya Zahra, Sarah Iqbal, Ayman Qahmash, Ijaz Hussain

**Affiliations:** 1 Department of Computer and Information Sciences, PIEAS, Lehtrar Road, Nilore, Islamabad 45650, Pakistan; bscs2201@pieas.edu.pk (I.M.); bscs2238@pieas.edu.pk (Z.Z.); 2 Department of Artificial Intelligence, Faculty of Computer Science & Engineering, GIK Institute of Engineering Sciences and Technology, Topi 23640, Pakistan; sarah.iqbal@giki.edu.pk; 3 Informatics and Computer Systems Department, King Khalid University, Abha 62521, Saudi Arabia; a.qahmash@kku.edu.sa

**Keywords:** Electronic Health Records, topic modeling, clinical pathway analysis, patient trajectories, Systematic Literature Review, PRISMA

## Abstract

**Background**: Electronic Health Records (EHRs) are a rich source of clinical information used for patient monitoring, disease progression analysis, and treatment outcome assessment. However, their large-scale, heterogeneity, and temporal characteristics make them difficult to analyze. Topic modeling has emerged as an effective method to extract latent structures, detect disease characteristics, and trace patient trajectories in EHRs. Recent neural and transformer-based approaches such as BERTopic has significantly improved coherence, scalability, and domain adaptability compared to earlier probabilistic models. **Methods**: This Systematic Literature Review (SLR) examines topic modeling and its variants applied to EHR data over the past decade. We follow the Preferred Reporting Items for Systematic Reviews and Meta-Analyses (PRISMA) framework to identify, screen, and select relevant studies. The reviewed techniques span traditional probabilistic models, neural embedding-based methods, and temporal extensions designed for pathway and sequence modeling in clinical data. **Results**: The synthesis covers trends in publication patterns, dataset usage, application domains, and methodological contributions. The reviewed literature demonstrates strengths across different modeling families, while also highlighting challenges related to scalability, interpretability, temporal complexity, and privacy when analyzing large-scale EHRs. **Conclusions**: Topic modeling continues to play a central role in understanding temporal patterns and latent structures in EHRs. This review also outlines future possibilities for integrating topic modeling with Agentic AI and large language models to enhance clinical decision-making. Overall, this SLR provides researchers and practitioners with a consolidated foundation on temporal topic modeling in EHRs and its potential to advance data-driven healthcare.

## 1. Introduction

The growing trend in the digitization of healthcare has resulted in an unprecedented accumulation of Electronic Health Records (EHRs), which capture multifaceted aspects of patient care such as diagnoses, medications, laboratory tests, and clinical notes [1]. These EHRs have huge potential to fuel data-driven healthcare by permitting insights into disease trajectory, treatment response, and clinical pathways. They are complex in nature, high-dimensional and heterogeneously structured, and naturally occupy long temporal ranges. This makes it extremely difficult for classic statistical methods to extract useful patterns from such data at scale [2,3].

Topic modeling has emerged as a powerful set of methods that can identify hidden structures in EHRs [4,5,6]. First developed in the context of natural language processing, topic models were brought to healthcare to identify hidden disease phenotypes, determine patient subgroups, and map longitudinal clinical trajectories [7]. With time, research evolved from initial matrix and tensor factorization techniques to probabilistic models, like Latent Dirichlet Allocation (LDA) that would be able to model multi-modal healthcare data [5]. More recently, topic modeling has been extended to cover larger datasets using neural embedding-based models like neural variational models and transformer-based approaches [8]. Recently introduced temporal extensions of topic models have also allowed for the detection of changing clinical patterns, and hence are especially useful for pathway mining and longitudinal patient analysis [9] over time. This trajectory over time represents an ongoing effort to increase scalability, interpretability, and practical applicability to complex real-world healthcare environments [10].

The increasingly large body of work has put these various approaches to a variety of healthcare tasks, such as phenotype discovery, comorbidity analysis, patient risk stratification, and clinical decision support. Consistent with this advancement, the literature remains fragmented, with studies differing in methodology, datasets, and application scope. There is a trilemma involving scalability, clinical interpretability, and temporal depth in the field. As a result, there is still no agreement on the paradigms that are most appropriate for particular data forms or clinical operations. Moreover, as the field moves toward decentralized and privacy-sensitive settings, the trade-offs between the complexity of the model and the control over the data remain poorly understood. Thus, a unified SLR is needed to chart the field, distill findings, and underscore gaps that are still open for investigation. This SLR is unique in presenting a multidimensional comparative model that compares topic modeling paradigms to the EHR trilemma, which is an inherent trade-off between scalability, clinical interpretability, and temporal depth. In contrast to past surveys, we also analyzed which technique was most suitable for each specific dataset type. Also, our framework provides a comprehensive analysis regarding the performance of each method for topic modeling. In addition, our analysis focuses on the integration of Agentic AI with large language models (LLMs) by taking into consideration the gap between latent topic discovery and autonomous and clinician-centered decision support.

This SLR seeks to offer such a consolidation through the systematic examination of 79 out of 557 full-length peer-reviewed articles published between 2015 and 2025, which utilize topic modeling for the analysis of EHRs, till 30th September, 2025. Through this effort, we present a systematic analysis of methodological advancements, trend in publications, usage of datasets, and clinical applications. Furthermore, we synthesize strengths, weaknesses, future research directions, and lessons learned across topic modeling paradigms.

The primary contributions of this survey are as follows:

(i) We provide an in-depth taxonomy of topic modeling techniques used in EHRs, covering probabilistic methods, matrix factorization methods, neural methods, transfer learning methods, and temporal extensions.
(ii) We offer an extensive analysis of research findings, such as dataset used, evaluation measures, topic modeling technique, strengths, and limitations of these studies.
(iii) We review the existing challenges to apply topic modeling in healthcare, e.g., scalability, interpretability, and data privacy, and present promising avenues for future research, including the integration of Agentic AI and large language models into clinical pathway analysis.

## 2. Methodology

**Figure 1:**
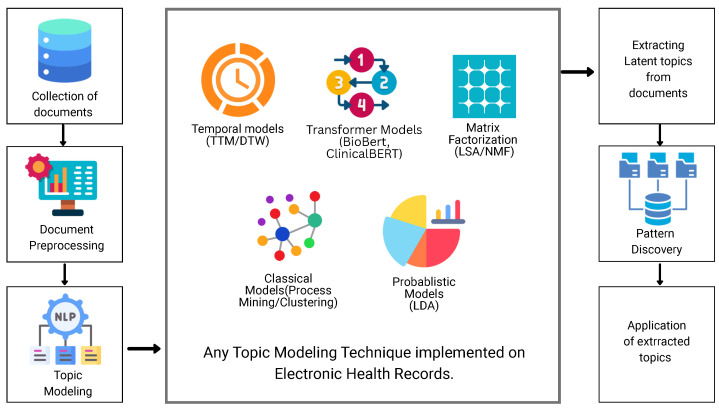
Architecture of the survey from raw EHRs to topic modeling techniques, pattern discovery, and clinical applications of extracted topics.

This SLR aims at systematically reviewing the relevant literature of topic modeling techniques applied to EHRs using the PRISMA framework, and its protocol has been registered in the Open Science Framework database with the following DOI: 10.17605/OSF.IO/URPYG. We describe the approach used to identify, screen, and evaluate research for this SLR, including how search strings are formed, which keywords are employed, the search approach taken to retrieve the articles, the classification structure chosen, the distribution of papers and datasets, and the criteria used to compare the selected articles. We selected our desired literature content according to the strategy shown in Figure 1, which shows the layered architecture of topic modeling applied to EHRs for pattern discovery and clinical applications. The documents created from the EHRs should be preprocessed to be fed into some topic modeling technique such as Latent Dirichlet Allocation, temporal topic models or Non-negative Matrix Factorization, etc. The topic modeling technique extracts the topics from the documents, which can be used for further potential applications such as phenotyping clinical pathways.

### 2.1. Defining Research Questions

This SLR investigates the following research aspects: (a) What is the taxonomy of topic modeling techniques applied in EHRs? (b) What datasets are available for the research and development of EHR topic modeling techniques? (c) How are diverse topic modeling techniques applied in EHRs? (d) What are the research gaps, challenges, and future research directions in EHR topic modeling?

The objective of this SLR is to address a set of focused research questions that guide our analysis of topic modeling in EHRs:

- RQ1: What are the predominant topic modeling approaches for EHRs and how can they be compared to each other?
- RQ2: How have topic modeling methods evolved over time in the context of EHRs?
- RQ3: What are the strengths and weaknesses of the existing studies in EHR systems?
- RQ4: What are the challenges and future research directions in the field of topic modeling for EHRs?

### 2.2. Selecting Databases

Based on the formulated research questions, a search query was used in both the bibliographic databases, i.e., Scopus and Google Scholar. The choice of Scopus and Google Scholar was to have a wide capture of the multidisciplinary intersection of clinical medicine and computer science. Scopus, a formal repository, was used as the main one because of its better indexing of peer-reviewed journals in data mining and medical informatics. Google Scholar was included as a critical secondary source to capture the literature, conference papers, and articles available as early access, which may not yet be entirely indexed in curated repositories. Although some specific databases like PubMed and IEEE Xplore were first searched, a sensitivity analysis performed at the preliminary stage revealed that their results were fully covered by Scopus and Google Scholar. Treating these two platforms as a comprehensive union allowed us to have a high recall rate and remove duplicates that were redundant, so that the 79 articles selected constituted the entire range of the current state-of-the-art literature.

Each database returned different numbers of articles. The search was performed in September, 2025. An objective set of inclusion and exclusion criteria as shown in Table 1 was defined for retrieving articles. These criteria reflect the main objective of this SLR and help us to thematically focus on the most current and relevant articles to see how diverse topic modeling techniques are being used in the healthcare domain using EHRs, and to focus on available datasets in healthcare and the relevant performance metrics used.

### 2.3. Formulating Search Terms

**Figure 2:**
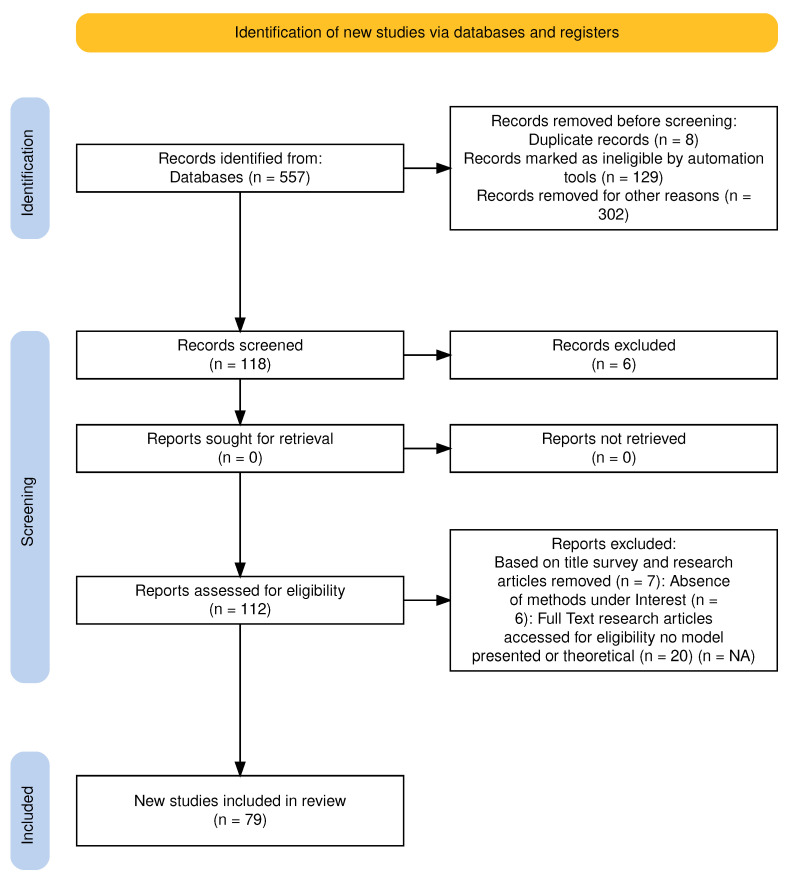
PRISMA format diagram of the presented systematic review selection process.

To find the answers for the research questions posed in Section 1, relevant articles for topic modeling in EHRs were collected using the online databases Scopus (https://www.scopus.com/) and Google Scholar (https://scholar.google.com), as mentioned in Section 2.2. The PRISMA diagram in Figure 2 illustrates the selection and screening process for the articles included in this SLR.

Starting with “topic modeling” as the primary keyword. From this initial collection, we identified and chose more keywords to further expand the initial set of articles. Examples of these keywords include “topic modeling in EHRs”, “temporal topic modeling”, “clinical pathway analysis using NLP”, “EHR analysis using NLP”, “role of topic modeling in healthcare”, “transformers for topic modeling”, “classical approaches for EHR analysis”, and “Topic modeling in clinical pathway analysis”, according to the examples of initial search performed using the search strings listed in Table 2.

### 2.4. Applying Inclusion and Exclusion Criteria and Synthesizing Articles

In the identification phase of PRISMA, 557 articles were initially retrieved from both the databases. Using AI automation tools to remove the articles not in our scope, 129 articles were marked ineligible. We checked titles and abstracts and filtered an additional 302 articles on the basis of the exclusion criteria, like articles older than 2015 to avoid outdated knowledge, articles not written in English, type of study, not using EHRs, irrelevant content, and non-accessible full texts [11,12,13]. Eight article records were found to be duplicates. A total of 118 records were selected in the screening phase, where we studied whole articles to decide whether it should be included or not. We further filtered gray literature that includes technical reports, white papers, periodical statistical reports, etc., usually published by governmental institutions. Some studies did not use any topic models and only included theoretical work. We believe that such articles are not relevant to the scope of this SLR. At the end of the screening phase, as illustrated in Figure 2, a total of 118 papers were initially identified, of which 79 papers were finalized for detailed analysis in this SLR. The first three authors agreed to include only these articles. In addition to relevance, other factors were considered, such as completeness in terms of task definition, description of the proposed model or method, and presentation of results.

### 2.5. Classifying Topic Modeling Techniques for EHR

**Figure 3:**
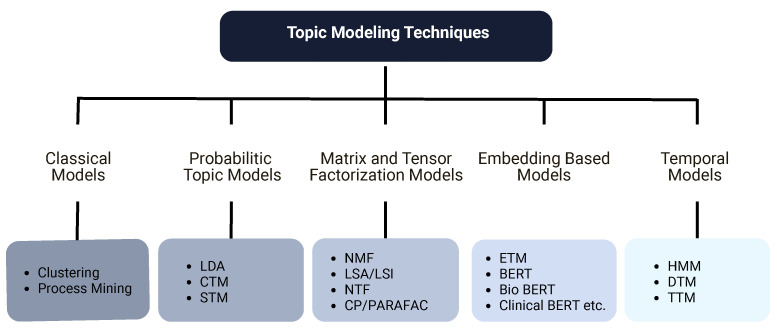
Taxonomy of topic modeling techniques applied to EHRs.

Based on the methodological families of topic modeling techniques employed to EHRs, as seen in Figure 3, we classified the selected techniques into the following five disjoint classes with associated technologies. Classical models such as clustering and process mining offer straightforward yet powerful ways to arrange highly dimensional EHR information first. Probabilistic topic models include LDA, CTM, STM, and clustering-based derivatives like K-means. These models provide clear statistical basis for hidden topic identification. Matrix employing tensor factorization approaches, the third family, expands dimensionality reduction by capturing multi-way linkages across patients, visits, and temporal slices. Using contextualized embeddings, group four, ETM and BERT, finds semantically rich patterns based on distributed representations using embedding-based models. Finally, temporal models particularly match the study of patient trajectories and clinical pathways because they explicitly represent topic evolution.

### 2.6. Publication and Dataset Distribution

To better understand the characteristics of the selected articles, we investigated the distribution of publications across several classifications, such as methods employed, type of publication, location, temporal patterns, and geographical distribution. These distributions explain the growth and scope of research in topic modeling techniques for EHRs.

**Figure 4:**
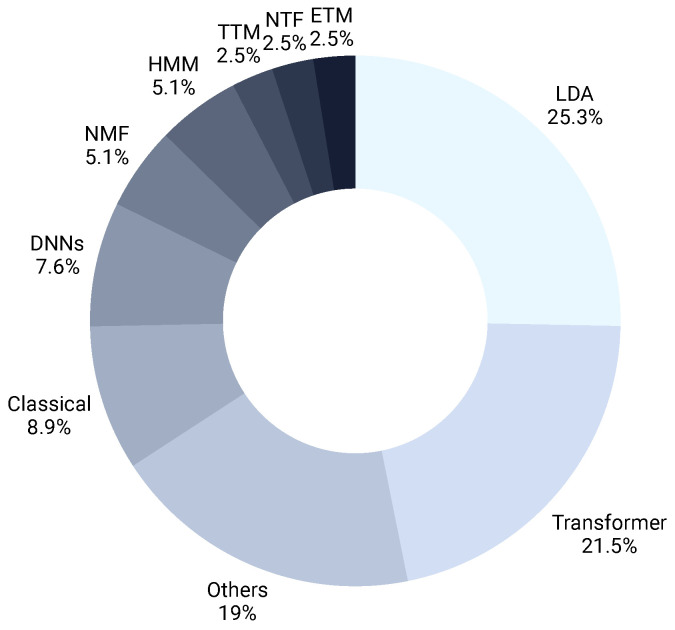
Distribution of selected papers across different topic modeling techniques.

Figure 4 highlights the relative share of each methodological family in this SLR literature. This breakdown is essential to assess which approaches dominate current research, and where underexplored opportunities remain. LDA was the leading choice of scientist in this field with 20 articles, whereas TTM, NTF, and ETM each were applied only two times. Two-thirds of the articles applied LDA, transformer variants, and LSA/CTM. Other articles used clustering, process mining, deep neural network, and NMF. A strong dominance of these LDA-based approaches signify their maturity, interpretability, and ease of use. In contrast, the sparsely explored embedding-based approaches highlight their limitations with respect to increased model complexity and data preprocessing challenges.

**Figure 5:**
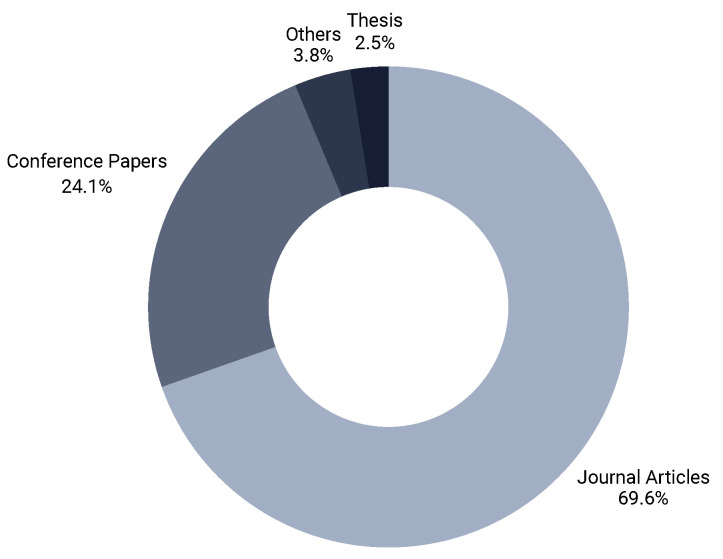
Number of papers by publication type (journal vs. conference).

Similarly, Figure 5 shows the distribution of the articles into different types of publication, e.g., journals, conferences, thesis reports, and book chapters, reflecting the balance between mature contributions and emerging explorations. About seventy percent of the publications were journal articles while a few were theses, and only 2 of the 79 were about topic modeling in EHRs, which shows a considerable research gap for potential researchers in this promising field.

**Figure 6:**
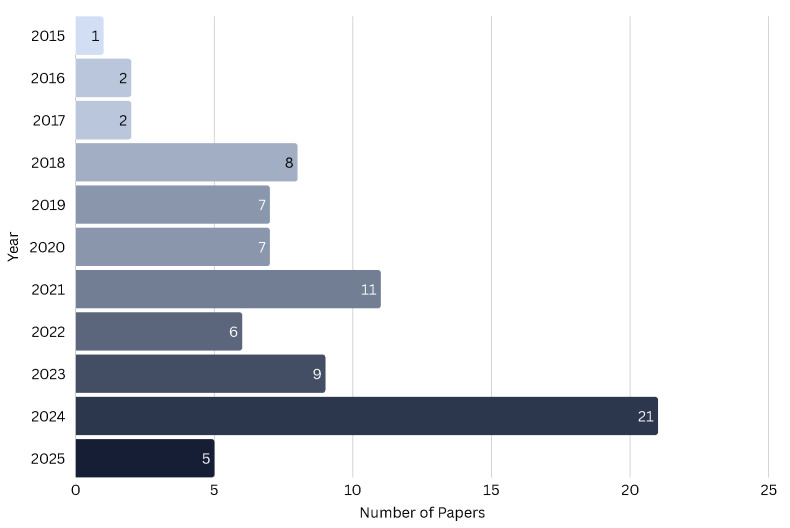
Publication trend of selected papers over time (2015–2025).

**Figure 7:**
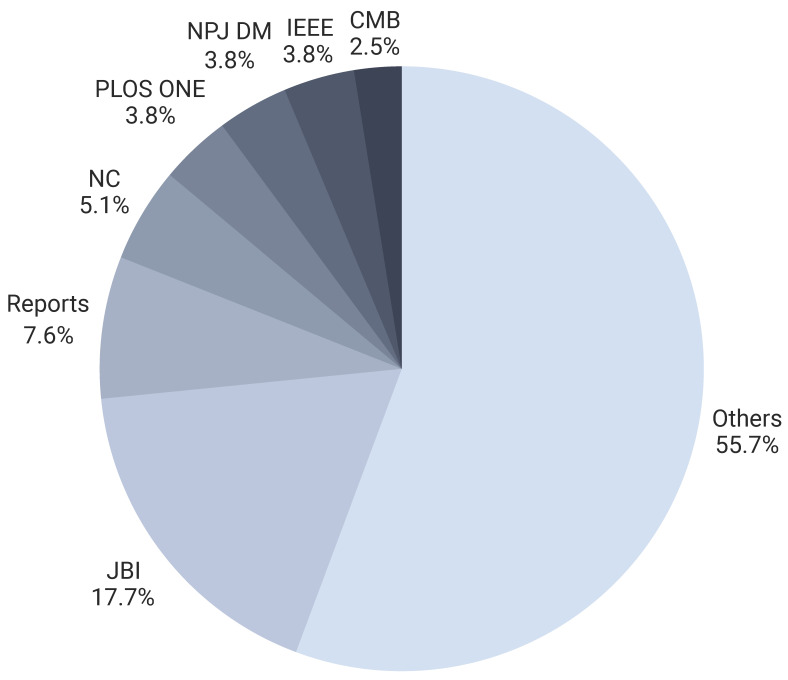
Distribution of selected papers across major publication venues.

Figure 6 depicts the year-wise publication trend, illustrating the growing momentum of this field in recent years. Figure 7 identifies the most prominent journals and publishers, highlighting where this research is concentrated and which venues are shaping the discourse.

**Figure 8:**
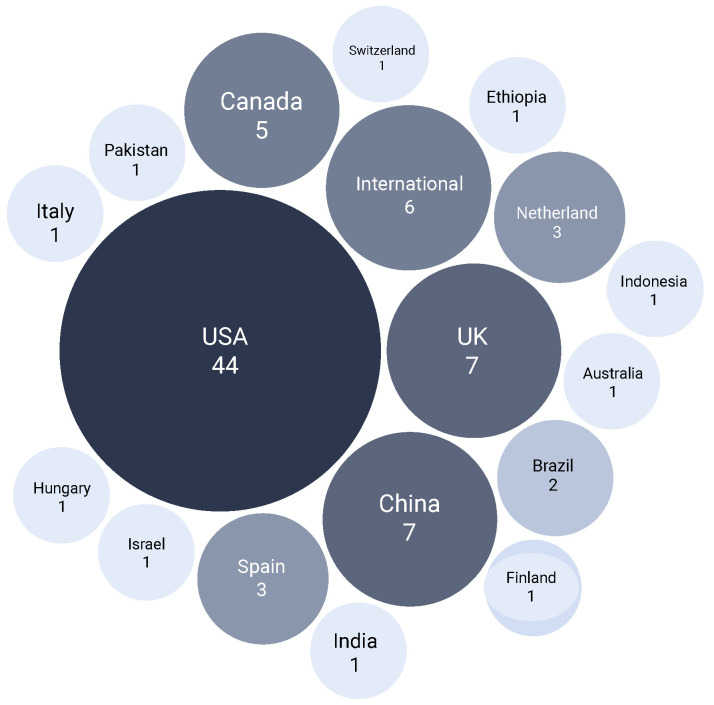
Geographic distribution of publications by country.

Figure 8 shows the geographic spread of contributions, reflecting global interest and the role of different healthcare systems in driving research priorities.

Table 3 summarizes the three primary dataset categories used across the 79 selected studies. The MIMIC dataset is used in 15 papers, UK Biobank dataset in 2 papers, and the remaining studies use various proprietary hospital EHRs aggregated here. The table also presents an analysis on the relationship between dataset type and the model it is best suitable for. There is an evident match between the data modality and methodology: neural models are best applied to rich text benchmarks in MIMIC. Matrix factorization is most efficient in UK Biobank, i.e., the dataset with structured and genomic sequences, and temporal innovation is propelled by proprietary EHRs because of its longitudinal depth.

### 2.7. Comparison Criteria

In this SLR, we used four criteria concerning methodological design, evaluation strategies, temporal aspects, and reported limitations to compare and synthesize the studies.

Techniques Used:The topic modeling techniques are classified into five methodological families, e.g., classical models, probabilistic topic models, matrix and tensor factorization models, embedding-based neural models, and temporal extensions. This comparison supports identifying the main techniques in use, revealing methodological diversity, and tracing the evolution of topic modeling from traditional statistical methods to neural and hybrid methods.

Performance Metrics: Different studies utilize multiple evaluation metrics. Metrics that rely on probabilities, such as perplexity and coherence, are for assessing topic quality, while task-based metrics would entail a predictive or classification setting with measures, e.g., accuracy, F1-score, AUC, or clustering validity indices.

Temporal Dynamics Integration: A major distinguishing feature of the studies is whether they capture temporal information. This gives a guiding view concerning the evolving states of the disease that really suit pathway analyses or longitudinal explorations of EHR data.

Limitations and Challenges: Finally, we discuss the constraints admitted in the studied works, including scalability issues with big EHR datasets, challenges in modeling complicated temporal dependencies, privacy concerns about using hospital records, and the restricted interpretive capacity of sophisticated neural techniques. Recognizing these difficulties offers a balanced viewpoint and helps to shape future research directions.

## 3. Topic Modeling Methods for Electronic Health Records

**Figure 9:**
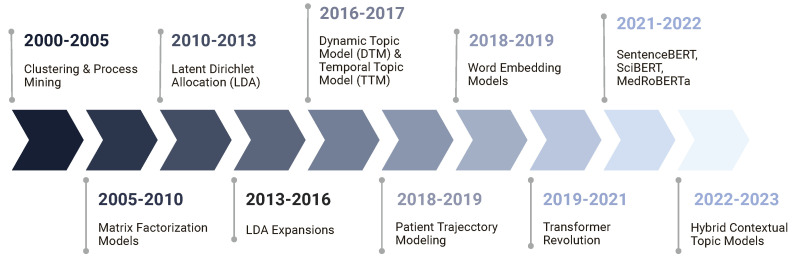
Timeline of key topic modeling methods in healthcare.

This section reviews the principal methodological families represented in the studies. The taxonomy used to organize these families is shown in Figure 3, and a historical perspective on method development is provided in Figure 9 [13], supported by the distribution of techniques used across the years as depicted by Figure 4 as well.

In the early 2000s, research was primarily driven by classical approaches such as clustering and process mining. These techniques focused on uncovering the patterns in textual data. After that, the research direction shifted towards matrix factorization techniques for topic modeling such as Non-negative Matrix Factorization (NMF) and Latent Semantic Analysis (LSA) techniques. These techniques enabled more structured and interpretable topic representations than the previous ones. During the course of the following decade, Latent Dirichlet Allocation and its extended versions, including the temporal models, were primary techniques for topic modeling. With the rise in transformers in 2017 and the years that followed, people have tried multiple approaches for topic modeling using transformer models to increase the semantics and contexts of their topics, reflecting the broader influence of deep learning on the field.

### 3.1. Classical Approaches

Classical approaches view patient records or document units as feature vectors, and attempt to uncover hidden groups via clustering or identify procedural processes using process mining techniques [14]. Common instantiations include k-means, hierarchical agglomerative clustering, density-based clustering, i.e., DBSCAN or HDBSCAN, and process mining methods such as alpha miner variations or heuristic miner and their more recent probabilistic or workflow-discovery extensions.

Technically, clustering algorithms work on an explicit representation $X\in R^{n\times d}$, whereby columns are TF–IDF, bag-of-codes, or embeddings, and each row is a document/patient attributes. K-means reduces within-cluster squared Euclidean distances.

$$\min_{C,\{\mu_{k}\}}\sum_{i=1}^{n}{∥x_{i}-\mu_{C(i)}∥}^{2},$$

Spectral or hierarchical clustering employs graph or linkage-based standards. Methods based on density search feature space for areas of high density and are insensitive to non-spherical clusters.

Process mining models work on event logs $L=\{\left(p_{i},\langle e_{i,1},\ldots ,e_{i,T_{i}}\rangle \right)\}$, where each trace is a time-ordered sequence of coded events; the goal is to deduce a process model, i.e., Petri net, transition graph, or probabilistic automaton, that accounts for observed behavior [15]. Noise filtering, frequency thresholds, and role or activity abstraction are frequently found in workflow discovery.

Usually applied following feature extraction in EHR applications, these techniques vectorized clinical notes, i.e., TF–IDF or embeddings, and encode structured events as categorical or temporal features. Clustering groups comparable topic mixtures, and process mining produces explicit pathways and transition probabilities for sequential studies, hence enhancing topic-based representations by offering cohort identification. Important hyperparameters included for process mining are number of clusters (*K*), distance metric, density thresholds, minimum support, and abstraction granularity.

### 3.2. Probabilistic Topic Modeling Approaches

Probabilistic topic models define documents or patient records as mixes over hidden themes and represent word generation via topic-specific word distributions. Using Dirichlet priors $\theta_{d}∼\mathrm{Dir}\left(\alpha \right)$ for document–topic proportions and $\phi_{k}∼\mathrm{Dir}\left(\beta \right)$ for topic–word distributions, the archetypal model, Latent Dirichlet Allocation (LDA), is drawn by first sampling a topic $z_{d,n}∼\mathrm{Categorical}\left(\theta_{d}\right)$, and then $w_{d,n}∼\mathrm{Categorical}\left(\phi_{z_{d,n}}\right)$. For large corpora, inference is usually carried out using collapsed Gibbs sampling, variational Bayes, or stochastic variational inference.

Correlated Topic Models (CTMs) capture topic correlations by replacing independent Dirichlet priors with logistic normal priors, and Structural Topic Models (STMs) use regression-style components to add document-level co-variates into topic prevalence and topical content. Supervised versions like sLDA or guided/seeded LDA introduce outcomes through generalized linear models, or supervise by labeling topic proportions [13].

EHR-specific instantiations usually use either clinical notes or concatenated notes per patient or code-sequences—documents per visit level codes as the input. Important modeling decisions include the unit of analysis, i.e., note-level, visit-level, or patient-level, vocabulary creation, i.e., words, clinical ideas, ICD/PheCodes, and their priors for sparsity and regularization. Essential hyperparameters include topic count *K*, Dirichlet concentration parameters α,β, and for STM or CTM, the form of covariates and their link functions. When issues are used as characteristics, evaluation employs intrinsic measurements such as topic coherence, perplexity, and downstream extrinsic activities, i.e., classification, clustering, and survival modeling.

### 3.3. Matrix and Tensor Factorization Approaches

Matrix factorization methods, e.g., Non-negative Matrix Factorization (NMF) and tensor decompositions generalize topic discovery to linear-algebraic latent factorization. NMF seeks non-negative matrices $W\in R_{\geq 0}^{n\times r}$ and $H\in R_{\geq 0}^{r\times d}$ such that X≈WH, where *X* is a term—either document or event-count matrix. Optimization is typically performed by alternating multiplicative updates or projected gradient methods minimizing an objective such as KL divergence or Frobenius norm.

Tensor methods—such as CP, PARAFAC, PARAFAC2, and Non-negative Tensor Factorization—extend factorization to multi-way arrays $X\in R^{I\times J\times K}$ and model X as a sum of rank-1 components $X\approx \sum_{r=1}^{R}a_{r}\circ b_{r}\circ c_{r}$ [16]. PARAFAC2 and related variants accommodate irregular mode sizes (e.g., varying visit counts per patient) and are therefore well suited for EHRs that exhibit irregular longitudinal structures. Optimization is commonly handled via alternating least-squares with non-negativity and sparsity constraints when required.

In practice, factorization approaches are used when the multi-aspect structure is explicit: patient × code × time tensors capture how latent factors (akin to topics) evolve across visits or cohorts. Factor matrices provide interpretable loadings: a factor’s code-loading vector resembles a topic’s word distribution, while the patient-loading vector indicates patient affiliation. Hyperparameters include rank *r*, sparsity regularization coefficients, and temporal smoothing penalties. Factorization methods are highly effective at capturing multi-way interactions, but they require careful initialization and regularization to avoid degeneracy and overfitting.

### 3.4. Embedding-Based and Neural Topic Models

Embedding-based approaches replace sparse bag-of-words representations with dense continuous vectors, either pretrained static embeddings, i.e., word2vec and GloVe, or contextualized transformer embeddings such as BERT-family models. Neural topic models such as the Embedding Topic Model (ETM) integrate word embeddings into the topic–word parameterization, often through neural networks that map embedding spaces to topic distributions. Contemporary pipelines, e.g., BERTopic, leverage sentence/document embeddings from transformers; this is followed by density-based clustering, i.e., HDBSCAN and class-based TF–IDF, to produce human-interpretable topic labels [17].

From a technical perspective, ETM parameterizes topic–word distributions as functions of word embeddings $v_{w}$ and topic embeddings $t_{k}$, where $\phi_{k,w}\propto \exp\left(t_{k}^{⊤}v_{w}\right)$; inference may proceed with amortized variational inference using neural encoders [18]. Transformer-based workflows embed each document *d* into vector $u_{d}$ and then apply clustering or mixture models to discover groups in embedding spaces; topics are generated by extracting representative words via class-based TF–IDF or by fitting lightweight probabilistic components on top of embeddings [19,20].

In EHRs, embedding-based methods improve semantic capture of clinical language—capturing abbreviations, handling negation, and enabling robust handling of short notes or fragmented text [21]. Implementation considerations include choice of embedding backbone like general BERT vs. ClinicalBERT, dimension reduction strategies for clustering, and techniques for producing human-interpretable topic labels from embeddings. Computational cost is higher than classical models, and reproducibility depends on embedding model versions and pretraining corpora.

### 3.5. Temporal Topic Modeling Approaches

Temporal models clearly describe how latent topics or states change over time [22]. Approaches in this category include Dynamic Topic Models (DTMs), topics-over-time, state-space extensions of topic models, Hidden Markov Models (HMMs) with topic emissions [23], and sequence models. They combine subject inference with RNNs or transformer-based temporal encoders. Mathematical formulas differ: DTMs apply temporal dynamics to topic parameters $\phi_{k,t}$ by means of state evolution equations, e.g., Gaussian random walks in the natural-parameter space, whereas HMM-based variants assume a latent discrete state sequence $s_{p,t}$ for patient *p*, having transition matrix *A* and state-conditional emission models which are perhaps by themselves topic distributions.

Temporal modeling has to deal with unequal sampling, variable-length patient histories, and censorship for EHR data. Among the realistic fixes are time-binning (also called fixed-width window), and irregular-time models such as continuous-time variations, and missingness accommodation via data imputation or model enhancement. Temporal models are mostly used for pathway mining, clinical trajectory exploration, and modeling onset progression of illnesses. Key decisions in execution include the temporal granularity, state-space dimension for HMMs, smoothing strength for DTMs, and whether subject parameters evolve smoothly or may show sudden changes such as topic birth or topic death. Inference can be computationally demanding, e.g., SVI for DTM and forward–backward recursions for HMMs; hence, reliable initialization is imperative to prevent subpar local optima.

## 4. Findings for the Topic Modeling Methods in EHRs

In this section, we present all the comparative findings we found according to our study of all mentioned methodologies. Table 4 defines the legend used across the comparative analysis tables, reflecting the claims made in each reviewed study.

### 4.1. Classical Techniques

Studies consisting of any of the classical techniques of clustering, classification, process/sequence mining are classified in this category. Classical techniques [2,8,24,25,26,27,28] highlight how important their continuing relevance is in identifying clinical subgroups and treatment pathways. As summarized in Table 5, consensus clustering and patient-similarity methods showed ongoing ability to stratify varied groups, including sepsis or chronic disease cohorts. Similarly, process mining systems like MEDCP and fuzzy process mining detected recognizable treatment flows and captured workflow variety among many hospitals. Particularly, these approaches earned high scores on interpretability, often validated by clinical experts, hence drawing interest in situations where honesty is crucial.

The tables also emphasize their limitations [29]: heavy dependence on coding quality, site-specific preprocessing, and lesser ability to generalize across universities. Even when combined with transformers, e.g., TransformEHR, conventional methods had higher computation costs and required expert-intensive configuration. The findings show that although clustering and process path mining and verification still rely on mining, it is progressively being used with modern representation learning. These methods are expected to extend beyond predictive constraints and scalability problems.

### 4.2. Probabilistic Topic Modeling Techniques

Table 6 illustrates the comparison of probabilistic topic modeling techniques, classified in this category on the basis of any probabilistic technique used, such as LDA or any of its non-temporal variant, such as CTM, ETM, BTM, etc., and highlights their function as the fundamental techniques for EHR analysis, and their ongoing flexibility to various clinical tasks [3,6,9,10,17,21,30,31,32,33,34,35,36,37,38,39,40,41,42,43,44,45,46,47,48,49,50,51,52,53]. Particularly for the exploratory research of clinical notes, coding data, and multi-modal EHRs, LDA and its Bayesian extensions still constitute the most widely utilized method. Consistently proving interpretability, these investigations helped to spot disease clusters, care themes, and phenotypic subtypes across environments, including MIMIC-III, dementia cohorts, and institutional cardiology databases. More modern models including MixEHR, MixEHR-S, MixEHR-Nest, and MixEHR-Guided extended the probabilistic approach to encompass multi-modal data, supervised results, and nested phenotypes. Likewise, specialized solutions like the Graph-Embedded Topic Model (G-ETM) and sequence-based probabilistic models emphasized Bayesian generative structures’ adaptability in integrating relational knowledge or sequential EHR dynamics. These techniques were very helpful for understanding phenotype detection, risk classification, outbreak detection, and supporting downstream prediction models overall.

Simultaneously, the results show continuous difficulties. Classical LDA approaches usually relied on qualitative expert labeling and proved sensitive to preprocessing and topic-count decisions, therefore restricting reproducibility. Although supervised or guided versions increased alignment with clinical outcomes, they brought complexity, reliance on curated priors, and expensive computational costs. Probabilistic models found problems with short-text collections, cross-institution generalizability, and management of temporal dependencies without clear extensions as well. Even hybrids including optimization techniques, e.g., Bayesian hyperparameter tuning, clustering integrations, and ChatGPT-3.5-assisted interpretation, underlined both the continuing relevance and natural limits of probabilistic models. Generally, these findings indicate that probabilistic models still anchor the field owing to their interpretability and theoretical basis [13], yet their long-term value progressively rests upon deliberate improvement with multi-modal, relational, and temporal elements to satisfy modern EHR analysis criteria.

### 4.3. Matrix and Tensor Factorization Techniques

Studies have been classified in this category on the basis of the presence of any factorization method such as NTF, NMF, LSI, LSA or any of their variants. As the comparison in Table 7 shows, NMF and tensor-based models [23,29,54,55,56,57,58,59] have been particularly successful in identifying hidden phenotypes and multimorbidity patterns from organized EHRs [60]. While static and temporal features were successfully combined using PARAFAC2-based methods, constrained tensor factorization caught changing cardiovascular disease phenotypes, for example. These approaches always produced understandable factor loadings, therefore enabling the detection of important subgroups and temporal patterns. These evaluations often reduced multiple-testing risks and improved clinical coherence, therefore indicating great benefit for hypothesis generation.

Furthermore, the review identified several limitations associated with factorization methods. Their performance is much influenced by rank selection, sparsity, and preprocessing; they often struggle to integrate text data or erratic event series. Another often cited limitation was computational cost, especially for large temporal tensors. Matrix and tensor methods are still strong for structured, multi-aspect data even if they need painstaking parameter tuning and usually benefit from hybridization with other methods for boosting scalability and generalization.

### 4.4. Embedding-Based and Neural Topic Modeling Techniques

Any study that utilized embeddings, mainly transformer-based or other neural-based approaches have been classified in this category. The synthesis in Table 8 reveals the quick post-2020 development of embedding-based and neural methods [7,13,16,20,22,61,62,63,64,65,66,67,68,69,70,71,72,73,74,75,76,77,78]; recent research shows improved performance of transformer-based and deep representational models in specific comparative studies [19]. Techniques like Med-BERT, BEHRT, and ExBEHRT showed clear improvements in predictive accuracy and phenotyping quality especially in multi-modal and longitudinal EHRs, complementing the early attempts toward generative models, as well as the hybrid approaches that merged EMRs2CSP, GPT-style Foresight, and LLM-assisted clinical route modeling.

However, the tables also show major challenges: high computation requirements, constrained interpretability, and data access problems usually limit repeatability across sites. Many transformer-based models also need a lot of pretraining materials, hence lowering their worth in resource-poor conditions. Still, these methods represent the cutting edge approach as there is strong evidence that there is growing research interest in embedding-rich and sequence-aware methods for temporally driven EHR modeling.

### 4.5. Temporal Models

The studies addressing the techniques incorporating any temporal-based topic modeling such as TTM, HMM, etc., are classified in this category. The methods of temporal modeling [5,79,80,81,82,83,84] underlined in the comparison Table 9 stress their relevance for EHRs in the study of patient trajectories, disease progression, and pathway development. Probabilistic generative strong abilities for managing structured sequences, modeling comorbidity evolution, and producing interpretable latent subtypes were found in latent-state models and HMM-based methods [5]. While extensions such as latent treatment topic models supported customized pathways, HMMs applied to co-occurrence patterns specifically captured temporal structures while keeping computational feasibility. Forecasters and next-step clinical decision support. By specifically following the change in topic incidence over time, temporal topic models like DTM and TTM provided new insights by revealing emerging risk elements and therapy flows. For instance, applications to GP notes found pre-fall incident signals by changing topic trajectories; national-scale databases like N3C allowed for the uncovering of temporal evolution for millions of patients showing long-COVID traits.

Simultaneously, the constraints on these techniques are both constant and rather great. Many models depend on solid simplifying assumptions, for instance, Markov independence or discrete latent states, which may oversimplify the complex temporal dependencies inherent in longitudinal EHR data. Results were highly sensitive to preprocessing choices, including window size and temporal aggregation. Furthermore, the issues of missing data or aberrant sampling presented additional difficulties. Furthermore, in temporal extensions, computational demands rose significantly as opposed to static models. Though temporal methods are essential for pathway mining and illness development modeling, these results show that their dependability relies much on data quality and pretreatment thoroughness. Hybridizing temporal topic models with continuous-time representations or flexible neural sequence encoders could enable more accurate modeling of patient journeys and so overcome these limitations.

### 4.6. Hybrid Models

The techniques that used any combination of the abovementioned techniques, such as probabilistic and embedding-based approaches or classical process mining with transformers, are classified as hybrid. The hybrid approach [4,18,85,86] comparison in Table 10 reveals the growing tendency to meld traditional topic modeling with supporting machine learning techniques in an effort to balance interpretability and predictive accuracy. Methods like FKLSA (Fuzzy K-Means + LSA + PCA) improved topic accuracy and stability on noisy corpora by means of clustering and dimensionality reduction, therefore showing benefits on conventional baselines like straight LDA or LSA [38]. A recent study revealing the benefits of integrating LDA with BiLSTM designs discovered the benefits of combining readable latent themes with temporal sequence modeling. Stronger adherence to better predictive indicators like AUC, accuracy, and cost/time efficiency, supplied by these hybrid systems showed that fusion models can directly optimize healthcare systems.

The results also highlight creative extensions like KG-TM, which pairs knowledge graph embeddings with probabilistic topic modeling. By anchoring latent themes to curated medical ontologies, this approach enhanced topic coherence and enabled ontology-guided phenotyping [36], hence producing more clinically relevant latent themes. Through these hybrid methods, more modeling layers should increase the temporal, semantic, or relational capacity of topic models while maintaining their human interpretability. There are still several limitations, however: noisy topic inputs may propagate mistakes into the hybrid system; knowledge graph models need considerable curation and mapping Work; and tuning the interaction between elements sometimes proves to be tough. Although cross-institution generalizability and robustness still need improvement, these results imply a good middle ground in hybrid models.

## 5. Challenges and Future Directions

Even though topic modeling has proven to be an effective approach to obtain observations from EHRs, many cross-cutting problems still obstruct its full use in clinical practice [13]. Every one of these challenges offers possibilities for more research: scalability, interpretability, temporal modeling, integration with new artificial intelligence techniques, and privacy. Table 11 provides a systematic comparison of how these problems impact each of the topic modeling techniques discussed according to the analysis.

Several probabilistic and neural topic modeling methods investigated in this SLR show promising performance on standard datasets but struggle to scale when applied to real-world environment settings as EHR databases contain millions of entries. At the same time, interpretability of buried subjects remains a usual concern. Correct models using these techniques cannot be included in decision support without clinically significant depictions. For guaranteed clinical utility, future studies must therefore focus on developing scalable architectures that keep interpretability by combining human-in-the-loop validation with efficient variational inference [20].

Many temporal additions to topic modeling have enabled researchers to identify changing disease patterns and patient journeys. Still, there is difficulty in modeling the full temporal richness of EHRs. The reviewed works imply that better grasping of complex clinical pathways demands hybrid methods combining temporal topic models with sequential learning algorithms like recurrent or attention-based systems [8].

None of the examined studies explicitly link temporal topic modeling with agentic systems. So, future research could look at how LLMs could be used with topic modeling results for natural language. Recent advances in LLMs and the development of Agentic AI offer new possibilities to improve traditional topic modeling approaches [87,88,89] by extending their outputs beyond static analysis toward dynamic interpretation, interaction, and decision support. While current studies primarily employ topic modeling to identify latent themes, LLMs could be a powerful tool to add context, summary, and translation of these topics into clinically meaningful narratives which in turn can significantly improve interpretability and usability for healthcare professionals. There is clearly a route towards the development of interactive, flexible, and understandable clinical agents that could dynamically manage topic modeling workflows, validate topic evolution over time, cross-check findings against clinical guidelines, and present insights in clinician-friendly formats like natural language summaries of disease trajectories or treatment pathways. Furthermore, agentic systems could monitor incoming clinical data streams in a proactive manner, trigger re-analysis when topic shifts are detected, and support clinicians through early warnings and evidence-based suggestions. While these capabilities have not yet been realized in practice, they represent a promising avenue for advancing explainable, scalable, and clinician-centered decision support systems in healthcare.

A common challenge with patient data is the privacy concerns. EHR data are also very sensitive; several of the articles examined rely on few, publicly available datasets like MIMIC-III [36]. This restricts the range of assessments and so compromises the generalizability of the findings. Federated learning and privacy-preserving technologies provide promising ways to scale topic modeling across organizations without exposing raw patient information [83]. Wide clinical adoption will rely on addressing fairness, data governance, and repeatability in federated topic modeling.

## 6. Conclusions

Although EHRs provide great promise for promoting data-driven healthcare, their complexity, scope, and temporal depth make analysis challenging. An efficient means to find hidden patterns, group patients, and trace trajectories has emerged through topic modeling. Using the PRISMA framework, this SLR examined 79 publications between 2015 and 2025. We surveyed publication trends, database use, and application fields by means of a taxonomy, including probabilistic, matrix factorization, neural, temporal, and hybrid approaches. Our synthesis draws attention to ongoing hurdles while also stressing methodical advancements in data-driven healthcare analysis.

The most notable discovery of the analysis is the unsolved trade-off between interpretability and scalability. Although contemporary models represent temporal depth, clinician trust is hampered by their black-box character. As of now, no single strategy meets every need for real-time deployment. There is an urgent need for clinician-centered agentic systems which must replace pure optimization in the field. In order to ensure that models are naturally built for human-in-the-loop validation, the focus is on developing hybrid frameworks where LLMs convert complicated subjects into practical narratives. Looking ahead, significant opportunities lie in creating privacy-preserving, transparent, efficient, clinically based models and in connecting topic modeling with Agentic AI and LLMs to produce more engaging and useful clinical resources. This SLR offers practitioners and researchers a consolidated reference and a road map for promoting temporal topic modeling in healthcare analysis.

## Figures and Tables

**Table 1:** Inclusion and exclusion criteria for articles in this SLR.

Inclusion Criteria
Articles on topic modeling, topic modeling using Electronic Health Records, topic modeling in clinical pathway analysis, topic modeling in healthcare, latent topic discovery in healthcare
Articles that use EHR data
Articles published in conferences, journals, and workshops
Articles published from 2015 to 2025
Articles in English language
**Exclusion Criteria**
Articles not about topic modeling in healthcare
Theoretical papers without empirical methodology or metrics
Gray literature (not published in any reputable venue or not peer-reviewed)
Papers published before 2015
Publications not in English

**Table 2:** Systematic search strategy and boolean logic.

Category	Keywords/Terms
Target Domain (A)	“Electronic Health Records”, “EHR”, “Clinical Notes”, “Structured Records”
Core Method (B)	“Topic Model*”, “Latent Dirichlet Allocation”, “LDA”, “NMF”, “Neural Topic Model”
Technical Focus (C)	“Temporal”, “Longitudinal”, “Sequential”, “Transformers”, “LLM”, “Agentic”
Application (D)	“Clinical Pathway”, “Phenotyping”, “Risk Stratification”, “Comorbidity”
Full Boolean String	(A) AND (B) AND (C OR D)
Filters Applied	Date: 2015–2025; Language: English; Document Type: Article, Conference Paper

**Table 3:** Combined view of dataset categories and their methodological interactions.

Dataset	Availability	Modality	Clinical Domain	Country	Dominant Techniques	Methodological Interaction
MIMIC	Public	Notes + structured	Critical care/ICU	USA	Probabilistic, Temporal, Neural-based Approaches	Ideal for NLP-heavy models extracting semantics from dense notes.
UK Biobank	Restricted	Structured + genomic	Population-scale studies	UK	Matrix Factorization, Embedding	Sparse categorical matrices favor dimensionality reduction.
Proprietary EHRs	Restricted/ Proprietary	Multi-modal (Notes, codes, labs)	Multiple domains	Multiple	Temporal, Embedding/Neural-based Approaches	Fragmented, longitudinal events necessitate temporal and neural layers.

**Table 4:** Legend of symbols and rating scales used in the comparative analysis tables.

Metric/Symbol	**★**	★★	★★★
Sensitivity	Highly sensitive	Moderately sensitive	Low sensitivity
Computation Cost	High cost	Moderate cost	Low cost
Complexity	High complexity	Moderate complexity	Low complexity
Scalability	Low scalability	Moderate scalability	High scalability
**Symbol**	**Meaning**		
✓	Included/Addressed:	Present in the proposed framework/model	
✗	Not Included/Absent:	Not addressed or missing in the model	

**Table 5:** Comparative analysis of classical approaches for topic modeling.

Ref. No.	Year	Technique	Data Modality	Sequence Discovery	Rule-Based/ Heuristic	Reliance on Clinical Ontology	Qualitative Evaluation	Generalizability	Discrete Patient Clusters Discovery	Coding/Log Quality Dependency
[24]	2019	Consensus Clustering	Structured Only	✗	✗	✗	✗	✗	✓	✗
[28]	2022	ICD TM (Code Co-occurrence)	Structured Only	✗	✓	✓	✗	✓	✗	✓
[26]	2022	MEDCP (Process Mining)	Structured Only	✓	✓	✓	✓	✗	✗	✓
[27]	2024	Patient Similarity + Process Mining	Structured Only	✓	✓	✓	✗	✗	✓	✓
[2]	2018	Patient Similarity + Pattern Mining	Structured Only	✓	✓	✓	✓	✗	✓	✓
[25]	2018	Sequence/Pattern Mining	Structured Only	✓	✓	✓	✓	✗	✗	✓
[8]	2024	TF-IDF + Supervised Classifier	Mixed (Text+Struct)	✗	✗	✗	✗	✗	✗	✗

**Table 6:** Comparative analysis of probabilistic modeling approaches for topic modeling.

Ref. No.	Year	Technique	Modeling Type	Data Modality	Data Limitation	Qualitative Evaluation	Quantitative Evaluation	Short/Noisy Text	Auxiliary Learning	Computation Cost
[21]	2020	LDA (Gensim)	Phenotype	Text Only	Note Quality	✓	✗	✗	✗	** ★★★ **
[49]	2023	LDA+text mining	Phenotype	Multi-Modal	Single Site	✓	✓	✗	✗	** ★★ **
[42]	2023	LDA+clustering	Phenotype	Multi-Modal	Preprocessing Sensitivity	✓	✓	✗	✗	** ★★ **
[48]	2021	MixEHR-S (Supervised BTM)	Prediction	Multi-Modal	Curated Inputs	✗	✓	✗	✓	**★**
[31]	2018	LDA	Thematic Analysis	Text Only	Note Quality	✓	✗	✗	✗	** ★★★ **
[44]	2024	MixEHR-Nest (Hier. Guided TM)	Phenotype	Multi-Modal	Ontology Mapping Quality	✓	✓	✗	✗	**★**
[35]	2023	G-ETM (Graph Embedded TM)	Phenotype	Multi-Modal	Graph Quality	✓	✓	✗	✗	**★**
[51]	2020	LDA+PDM clustering	Phenotype	Structured Only	Coding Bias	✓	✓	✗	✗	** ★★ **
[40]	2021	MNTM (Multi-Note TM)	Thematic Analysis	Text Only	Note Type Labels	✓	✓	✗	✗	** ★★ **
[38]	2019	LDA(feature for ITR)	Risk Strat.	Multi-Modal	Assumption Sensitive	✗	✓	✗	✓	** ★★ **
[30]	2018	LDA + supervised classification	Prediction	Text Only	Preprocessing Sensitivity	✗	✓	✗	✓	** ★★ **
[53]	2021	LDA (annotation for surveillance)	Surveillance	Text Only	Short-Text Sensitivity	✓	✓	✓	✗	** ★★★ **
[39]	2024	LDA (urology clinical text)	Thematic Analysis	Text Only	Small Dataset	✓	✓	✗	✗	** ★★★ **
[45]	2024	MixEHR-SurG (guided BTM+Cox)	Risk Strat.	Multi-Modal	PheCode Priors Quality	✗	✓	✗	✓	**★**
[9]	2021	LDA + Bayesian optimization	Thematic Analysis	Text Only	Metric-Dependent Performance	✗	✓	✗	✗	** ★★ **
[17]	2020	MixEHR (multi-modal BTM)	Phenotype	Multi-Modal	Discretization/Scaling	✓	✓	✗	✗	**★**
[47]	2022	LDA + feature-engineered ICD	Prediction	Text Only	Polysemy/Short Text	✗	✓	✓	✓	** ★★ **
[36]	2018	BTM + TF-IDF classifiers	Prediction	Text Only	Noisy Texts	✗	✓	✓	✓	** ★★ **
[52]	2022	Sequence latent variable model	Phenotype	Structured Only	Complex Inference	✓	✓	✗	✗	**★**
[41]	2022	CTM→STM (two-stage TM)	Thematic Analysis	Text Only	Note Completeness	✓	✓	✗	✗	** ★★ **
[6]	2015	PRSM (LDA-based for risk strat.)	Risk Strat.	Structured Only	Coded EHR Quality	✗	✓	✗	✓	** ★★ **
[37]	2024	LDA-style (clinical notes)	Thematic Analysis	Text Only	Note Quality/Variability	✓	✓	✗	✗	** ★★★ **
[10]	2024	LDA (low-resource language)	Thematic Analysis	Text Only	Low-Resource Language	✓	✓	✗	✗	** ★★★ **
[34]	2016	LDA-style (co-occurrence)	Thematic Analysis	Multi-Modal	Coding/Preprocessing	✓	✗	✗	✗	** ★★★ **
[46]	2017	Clinical LDA variant	Prediction	Structured Only	Institution-Specific Bias	✗	✓	✗	✓	** ★★★ **
[32]	2020	ETM (LDA+ supervised classifier)	Prediction	Text Only	LDA Weak for Short Text	✗	✓	✓	✓	** ★★ **
[43]	2022	MixEHR-Guided (semi-supervised)	Phenotype	Multi-Modal	Needs Strong Surrogates	✗	✓	✗	✗	**★**
[50]	2023	LDA+ChatGPT (TM+LLM)	Thematic Analysis	Text Only	LLM Hallucination	✓	✗	✗	✗	** ★★ **
[3]	2024	LDA/BERTopic + clustering	Phenotype	Multi-Modal	Text/Site Variation	✓	✓	✗	✗	** ★★ **
[33]	2021	LDA-like + supervised LOS pred.	Prediction	Text Only	Limited Generalizability	✗	✓	✗	✓	** ★★ **

**Table 7:** Comparative analysis of matrix and tensor factorization approaches for topic modeling.

Ref. No.	Year	Technique	Factorization Type	Data Modality	Temporal Dynamics	Subphenotype Utility	Handles Fuzziness	Computation Cost	Sensitivity to Data Sparsity	Model Extensibility
[54]	2019	Constrained NTF (CP/PARAFAC)	Tensor	Structured Only	✓	✓	✗	★	** ★ **	✓
[59]	2019	NMF (Phenome-Genome)	Matrix	Structured Only	✗	✗	✗	** ★★★ **	** ★★ **	✗
[58]	2019	LSI (SVD-based)	Matrix	Structured Only	✗	✓	✗	** ★★★ **	★	✗
[29]	2020	NMF (Temporal Topic Modeling)	Matrix	Text Only	✓	✗	✗	** ★★ **	** ★★ **	✗
[55]	2017	Constrained NTF	Tensor	Structured Only	✓	✓	✗	★	** ★★ **	✓
[57]	2020	NMF (Temporal Multimorbididty Phenotyping)	Matrix	Structured Only	✓	✗	✗	** ★★ **	★	✗
[23]	2020	PARAFAC2 + NMF (Joint TF)	Tensor	Structured Only	✓	✓	✗	★	** ★★ **	✓
[56]	2018	FLSA (Fuzzy LSA)	Matrix	Text Only	✗	✗	✓	** ★★ **	** ★★ **	✗

**Table 8:** Comparative analysis of transformer and deep neural network-based modeling approaches for topic modeling.

Ref. No.	Year	Technique	Core Architecture Type	Data Modality	Learning Strategy	Temporal Modeling	High Prediction Accuracy	Model Extensibility	Interpretability Mechanism	Computation Cost
[64]	2025	VaDeSCEHR	RNN/DNN/ VAE	Structured Codes Only	End-to-End Sup.	✓	✓	✗	Topic-Informed	★
[20]	2018	Deep DNN on FHIR	RNN/DNN/ VAE	Multi-Modal	End-to-End Sup.	✓	✓	✗	✗	★
[73]	2021	BERT-style embeddings	Transformer	Structured Codes Only	Self-Sup.	✓	✓	✓	✗	★
[22]	2025	Temporal deep rep + clustering	RNN/DNN/ VAE	Multi-Modal	Semi-Sup.	✓	✗	✗	Topic-Informed	★
[67]	2023	Transformer (BEHRT extension)	Transformer	Multi-Modal	End-to-End Sup.	✓	✓	✓	Feature Importance	★
[65]	2024	SMDBERT++	Transformer	Text Only	Semi-Sup.	✗	✓	✓	✗	★
[72]	2024	LATTE	Hybrid Deep	Structured Codes Only	Semi-Sup.	✓	✓	✓	Feature Importance	★
[76]	2024	SeDDLeR	RNN/DNN/ VAE	Structured Codes Only	Semi-Sup.	✓	✓	✓	✗	** ★ **
[63]	2021	Bidirectional Transformer	Transformer	Multi-Modal	Self-Sup.	✓	✓	✓	✗	★
[75]	2021	Hybrid: Hierarchical + Topic	Hybrid Deep	Multi-Modal	End-to-End Sup.	✗	✓	✗	Topic-Informed	★
[13]	2016	Stacked Denoising AE	RNN/DNN/ VAE	Structured Codes Only	Self-Sup.	✗	✓	✓	✗	** ★ **
[69]	2021	HCET (Topic-informed Hier.)	Hybrid Deep	Multi-Modal	End-to-End Sup.	✗	✓	✗	Topic-Informed	★
[62]	2021	BERT-style Transformer	Transformer	Structured Codes Only	Self-Sup.	✓	✓	✓	✗	★
[71]	2023	Hierarchical Transformer	Transformer	Multi-Modal	Self-Sup.	✓	✓	✓	✗	★
[68]	2024	GPT-style Transformer	Transformer	Multi-Modal	Self-Sup.	✓	✓	✓	✗	** ★ **
[16]	2019	ClinicalBERT	Transformer	Text Only	Self-Sup.	✗	✓	✓	✗	★
[70]	2024	HEART (Rel. Aware Trans.)	Transformer	Structured Codes Only	Self-Sup.	✓	✓	✓	✗	★
[66]	2025	LLM-assisted pathway extraction	Transformer	Multi-Modal	Semi-Sup.	✓	✗	✗	Topic-Informed	★
[74]	2021	Recurrent temporal model	RNN/DNN/ VAE	Structured Codes Only	End-to-End Sup.	✓	✓	✗	✗	** ★★ **
[77]	2023	Transformer encoder-decoder	Transformer	Structured Codes Only	Self-Sup.	✓	✓	✓	✗	** ★ **
[78]	2023	BERTopic	Transformer	Text Only	Unsup.	✗	✗	✗	Expert Validation	** ★★★ **
[7]	2025	LDA vs BERTopic	Transformer	Text Only	Unsup.	✗	✗	✓	Topic-Informed	★
[61]	2024	Seed-guided semi-supervised TM	RNN/DNN/ VAE	Text Only	Semi-Sup.	✗	✓	✗	Topic-Informed	** ★ **

**Table 9:** Comparative analysis of temporal approaches for topic modeling.

Ref. No.	Year	Technique	Core Temporal Mechanism	Data Modality	Modeling Focus	Interpretation Focus	Model Complexity	Scalability	Sensitivity to Binning/ Preprocessing
[5]	2024	Probabilistic Latent State Model	HMM/Latent State	Structured Sequences	Comorbidity	Comorbidity Linkage	** ★★ **	** ★★ **	★
[83]	2024	Hidden Markov Model (HMM)	HMM/Latent State	Structured Sequences	Phenotype	State Transition	** ★★★ **	** ★★ **	★
[79]	2024	Temporal Topic Model (Unsupervised LDA)	Time-Aware LDA	Structured Sequences	Phenotype	Topic Trajectory	** ★★ **	** ★★★ **	** ★ **
[80]	2021	Temporal Topic Model (LDA + time)	Time-Aware LDA	Structured Sequences	Treatment Flow	Topic Trajectory	** ★★ **	** ★★ **	★
[81]	2024	Hidden Markov Model (HMM)	HMM/Latent State	Structured Sequences	Progression	State Transition	** ★★★ **	** ★★ **	** ★★ **
[84]	2024	Dynamic Topic Modeling (DTM)	Dynamic Topic Model	Text	Evolution	Topic Trajectory	★	** ★★ **	★
[82]	2018	Latent Treatment Topic Model (HMM-like)	HMM/Latent State	Structured Sequences	Treatment Flow	State Transition	** ★★★ **	** ★★ **	** ★★ **

**Table 10:** Comparative analysis of hybrid approaches for topic modeling.

Ref. No.	Year	Technique	Hybridization Type	Interpretability Mechanism	Data Modality	Incorporates Deep Learning	Handles Temporal Data	Computation Cost	Sensitivity to Fusion	Predictive Accuracy
[4]	2019	FKLSA (Fuzzy K-Means + LSA + PCA)	Feature Fusion	Topic Coherence	Text	✗	✗	** ★★ **	** ★★ **	✗
[86]	2025	LDA + BiLSTM	Feature Fusion	Topic Coherence	Mixed	✓	✓	★	★	✓
[18]	2022	KG-TM (Probabilistic Topic + KG Embeddings)	Knowledge-Guided	Concept Linking	Structured	✗	✗	★	** ★★ **	✓
[85]	2023	Fuzzy Process Mining + Transformer	Feature Fusion	Feature Importance	Structured	✓	✓	★	** ★★ **	✓

**Table 11:** Systematic comparison of the impact of key challenges across topic modeling families.

Technique	Impact of Scalability	Impact of Interpretability	Impact of Temporal Complexity	Impact of Privacy Concerns
Classical Techniques	Scalable for mid-sized EHRs; efficiency drops with expanding clinical vocabularies.	Frequency-based transparency of reason; lacks multi-faceted topic depth.	Static architecture; unable to model sequential clinical visits or disease progression.	Requires centralized raw data; complicates patient de-identification.
Probabilistic Topic Modeling	Slow inference on large-scale EHRs; limits real-time decision support.	High clinical utility via word-probability distributions matching medical terminology.	Standard bag-of-words approach; ignores clinical event ordering.	Raw co-occurrence reliance; hinders decentralized/private implementation.
Matrix and Tensor Factorization	Mathematically scalable but high storage demand for sparse matrices.	Readable mathematical components; lacks probabilistic uncertainty modeling.	Supports time dimensions; complexity grows exponentially with visit frequency.	Operates on aggregated, de-identified matrices; avoids raw text exposure.
Embedding and Neural Topic Modeling	Highly robust; handles millions of records via mini-batch SGD.	Abstract latent spaces; difficult for clinicians to audit topic assignments.	Static by default; requires sequential layers (LSTM/Transformers) which increase training time.	Federated learning compatible; shares model weights without exposing raw EHR data.
Temporal Models	Increased complexity per time-step; long-term longitudinal analysis is significantly slower.	High utility for tracking disease evolution and patient journey trajectories.	Natively designed for temporal EHR richness; addresses a key SLR gap.	Identifiable sequential patterns; harder to anonymize than static records.
Hybrid Models	Dependent on complex components; combination overhead slows large-scale EHR processing.	Enhanced utility; leverages multiple methods to compensate for individual interpretability weaknesses.	Often acts as a temporal patch for static models; prone to sensitivity issues during feature fusion.	Variable; contingent on reliance upon raw text features versus processed embeddings.

## Data Availability

No new data were created or analyzed in this study.
